# Immunological effects of vitamin c and zinc on tilapia (Orechromis niloticus) exposed to cold water stress

**DOI:** 10.1371/journal.pone.0311078

**Published:** 2024-09-26

**Authors:** Ahmed Mustafa, Maryam Belavilas, Rumman Hossain, Israt Mishu

**Affiliations:** Department of Biological Sciences, Purdue University Fort Wayne, IN, United States of America; Tanta University Faculty of Agriculture, EGYPT

## Abstract

This study investigates the immunological and growth effects of Vitamin C and Zinc supplementation on Nile tilapia (*Oreochromis niloticus*) subjected to cold water stress. Nile tilapia fingerlings were housed in eight 20-gallon tanks at Purdue University, acclimated to 26 ± 2°C water conditions before the experiment. The tilapia was divided into groups with varying water temperatures and feed supplements: control fish in warm water, and experimental groups in cold water with increased levels of Vitamin C and Zinc. Stress was induced by lowering the water temperature to 15 ± 2°C in four tanks, while the remaining tanks were kept at the optimal growth temperature. Results demonstrated that Vitamin C and Zinc supplementation significantly enhanced immune response and muscle regeneration in cold-stressed tilapia, allowing them to achieve growth rates comparable to those of control fish in optimal warm water conditions. These findings highlight the potential benefits of combined Vitamin C and Zinc supplementation in improving the immune response and growth performance of tilapia under suboptimal temperature conditions.

## Introduction

Due to the growing global demand, Nile tilapia (*Oreochromis niloticus*) farming has expanded from tropical to temperate regions [1]. However, Nile tilapia is native to environments where water temperatures range between 82–86°F (28–30°C) [2]. As a result, at 72°F (22°C)—typical water temperatures in temperate climates—the growth rate of tilapia is three times slower than in tropical waters [3]. Feeding begins to decline at temperatures below 63°F (17°C), and the fish become more susceptible to diseases at 54°F (12°C) [2]. This intolerance to cooler temperatures is a significant factor limiting widespread outdoor farming of tilapia in colder regions of North America and Europe, necessitating expensive indoor facilities where water must be heated to maintain optimal growth temperatures [1].

Vitamin C is essential for growth, reproduction, development, and immune response in vertebrates, including fish [3]. It is also playing a crucial role in collagen synthesis, a key component of connective tissue and skin. Collagen formation is vital for wound healing and maintaining the structural integrity of tissues, especially under stress conditions like temperature fluctuations [3, 4]. Supplementation with Vitamin C has been shown to help tilapia cope with crowding stress and improve overall health [1]. Additionally, Vitamin C functions as an antioxidant, protecting cells from oxidative damage and supporting immune function by enhancing the activity of phagocytes, lymphocytes, and the overall response to pathogens [4–6]. Most fish cannot synthesize Vitamin C due to the lack of the enzyme L-gulonolactone oxidase, which is required for its biosynthesis, and making dietary supplementation necessary to prevent deficiency-related issues such as impaired growth, increased susceptibility to infections, and poor wound healing, thus it must be supplemented in commercial fish feeds. However, there is limited information about the optimal Vitamin C requirements in tilapia diets. Previous research has indicated variations in optimal concentrations, ranging from 79 mg/kg to 400–500 mg/kg of feed for tilapia fry and juveniles [5, 6]. Mature adult tilapia has been shown to prefer 420 mg/kg of Vitamin C in their diet [7]. We aimed to investigate the effects of increasing concentrations of Vitamin C supplementation in commercial fish feed on overall fish health. This study implemented an optimal concentration of 500 mg/kg of Vitamin C for control based on the previous study and co-supplementation experiments with Zinc.

Zinc is another essential micronutrient that enhances the immune system, immune response and physiological process in fish [8]. It is a catalytic component or structural constituent of various enzymatic systems, such as DNA and RNA polymerases, protein synthesis machinery, carbonic anhydrase, carboxypeptidase, and superoxide dismutase [8, 9]. Zinc is also involved in protein synthesis, the metabolism of nucleic acids, and the stabilization of cell membranes, thereby playing a significant role in immune function [10]. Furthermore, Zinc is crucial for wound healing as it contributes to the maintenance and repair of epithelial tissues by promoting cell proliferation and differentiation and its deficiency can lead to impaired growth and anorexia [10–12]. The absorption of Zinc occurs through the gastrointestinal tract and gills, with optimal dietary levels required to avoid deficiencies that can lead to growth retardation, immunosuppression, and increased mortality [13]. Carmo e Sa et al. [9] determined that the optimal concentration of Zn supplementation for weight gain in tilapia is 44.50 mg/kg. However, other studies [13, 14] have recommended a dosage of 30 mg/kg. Given that excessive Zn intake can result in toxicity, causing dissociation of gill epithelia and subsequent impairment of respiratory function [15], we decided to limit the Zn supplementation in this study fish feed to 30 mg/kg as recommended by [13, 14].

While studies have demonstrated the benefits of Vitamin C and Zinc separately, there is a lack of information on the combined effects of these nutrients [7–9]. Furthermore, the aforementioned studies on the separate benefits were conducted 20 to 30 years ago and did not investigate the simultaneous supplementation of two nutrients. Both nutrients are involved in tissue or wound healing through distinct mechanisms: Vitamin C aids in collagen development, while Zinc forms complexes with oxygen donors of nucleic acids, facilitating tissue repair. In this study, we investigated the effects of cold stress on tilapia when subjected to increased supplementation of Vitamin C, both alone and in combination with Zinc, in commercial fish feed. Our aim was to determine if these supplementation strategies could promote growth without adverse effects. Additionally, we assessed the impact of these treatments on wound healing in tilapia. The experiment consisted of 3 separate studies conducted over a period of 7 weeks each. In the first two studies, we supplemented Vitamin C and Zinc individually. In the third study, we are co-supplemented both ingredients. Throughout each study, we measured various parameters, including blood glucose levels, condition factors, spleen somatic indices (SSIs), and wound healing, to evaluate the effectiveness of Vitamin C and Zinc supplementation.

## Experimental method

### Acquisition and maintenance of fish

Nile tilapia fingerlings were acquired from AmeriCulture Inc., Animas, NM, and housed in eight 20-gallon (75.6 L) tanks containing dechlorinated water. The tanks were situated in the Department of Biology at Purdue University at Fort Wayne (PFW). The fish were randomly distributed among the tanks and allowed to acclimate to tank conditions for two weeks prior to the commencement of the experiment. At the start of the experiment, all eight tanks were maintained at an optimal growth temperature of 26 ± 2°C (80 ± 5°F) [16] using Visi-Therm Deluxe 100W heaters (Marineland, Blacksburg, VA) in the control tanks. The aquaria were equipped with Millennium 2000 filtration systems (The Fish Place, Lancaster, PA) to ensure proper filtration and aeration of the water. The fish were fed once daily to satiation during both the acclimation period and the study duration. They received commercial fish feed, Aquaculture Grower 400 (Gray Summit, MO), which served as the control feed throughout the study. The water parameters were kept within optimal ranges: dissolved oxygen between 4.5–8 mg/L, pH within 6–9, ammonia less than 0.25 mg/L, nitrate within 25–50 mg/L, and nitrite below 0.3 mg/L. All measured water chemistry parameters conformed to the specified ranges as outlined in [16]. The fish were exposed to a 12-hour light and 12-hour dark cycle during both the acclimation and study periods.

### Experimental design

The experimental feed groups received the control feed (CF) with three different supplementations: 500 mg/kg Vitamin C (VC), 30 mg/kg Zinc (Z), and a combination of 500 mg/kg Vitamin C and 30 mg/kg Zinc (CZ). This study was performed 3 times to evaluate the efficacy of different feed supplementation combinations: Vitamin C only, Zinc only, and Vitamin C combined with Zinc. During each study, fish were divided into the following groups in replicates: Control Feed Stressed (CFS), Control Feed Unstressed (CFU), Vitamin C Stressed (VCS), Vitamin C Unstressed (VCU), Zinc Stressed (ZS), Zinc Unstressed (ZU), Combined Stressed (VCZS), and Combined Unstressed (VCZU). The experimental design included randomization and the use of biological replicates, with multiple fish per treatment group across replicate tanks, to ensure the reliability and reproducibility of the results. Stress (S) was induced by lowering the water temperature in 4 of the tanks to 15 ± 2°C (60 ± 5°F) by removing the heaters. The remaining tanks were maintained at 26 ± 2°C (80 ± 5°F), forming the control feed and unstressed experimental feed groups.

Throughout the study, each fish tank was fed their respective treatment feed once daily to satiation. Six fish (three fish per tank x 2 replicates) were sampled at weeks 0, 1, 3, 5, and 7 during the study. The fish were netted and euthanized swiftly (within 2 minutes of capture) in a 1-liter solution of 200 mg of MS-222 (Sigma-Aldrich, USA), following the techniques described in Gerwick et al. [17], Halloway et al. [18], and Hossain and Mustafa [19].

All experimental fish were maintained and taken care of following the guidelines of Purdue University Fort Wayne and an approved animal care protocol of PACUC (Purdue Animal Care and Use Committee).

### Condition factor (K) calculation

The condition factor (K) was determined by measuring the length and weight of all the sampled fish after euthanization. The condition factor was calculated using the formula: $K=\left(Weight\times 100/Length^{3}\right)$ [20].

### Blood collection and glucose measurements

Blood samples for glucose measurements were drawn from the caudal vein using a heparinized syringe. Blood glucose concentrations were determined by placing a drop of blood from each fish onto a glucometer strip, which was then read using a standard glucometer (Precision Xtra, Abbott Laboratories; Abbott Park, IL, USA), as validated by Wedemeyer et al. [16] and Hossain et al. [21].

### Spleen somatic index (SSI)

Spleen somatic index (SSI) was calculated by removing the spleen from each sampled fish and weighing it individually on an electronic scale. The SSI was calculated using the formula: SSI=(spleenweight/bodyweight)×100[22].

### Wound healing assay

The phagocytic response was assessed using thioglycolate to elicit an immune response via macrophage cells [23]. At week 7 of each feed supplementation study, four fish per tank underwent a wound healing assay, following the methods described by Sobhana et al. [24]. This assay evaluated the body’s ability to recover from tissue damage. Fish were anesthetized by immersion in water containing MS-222 at a concentration of 50 mg/L. Subsequently, they were injected with 1 mL of thioglycolate using a 26-gauge syringe. The fish were then placed back into a recovery tank before returning to their original tanks. Sampling was conducted at 0-, 4-, 8-, and 24-hours post-injection (hpi), with the fish being placed in a 10% formalin solution (1 part formaldehyde and 9 parts PBS) at each time point. Control fish, which were not injected with thioglycolate, were also sampled. The injection site was excised and sent to the Purdue University Pathology Department in West Lafayette, IN, for histological sectioning and staining using a standard H&E protocol [25]. After tissue sectioning, mounting, and staining of the slides, they were examined under a microscope to quantify the number of phagocytic cells and assess the degree of tissue inflammation and repair [26].

### Statistical analyses

The data obtained from these experiments were analyzed using one-way analysis of variance (ANOVA) with Minitab (Minitab® Release 14) and Sigmaplot 12.0. For all data that were statistically significant (P < 0.05), a Tukey’s HSD test (post-ANOVA comparison of multiple means) was performed to determine differences between treatments. The data are presented as means ± standard errors of the means (SEM).

## Results

**Fig 1:**
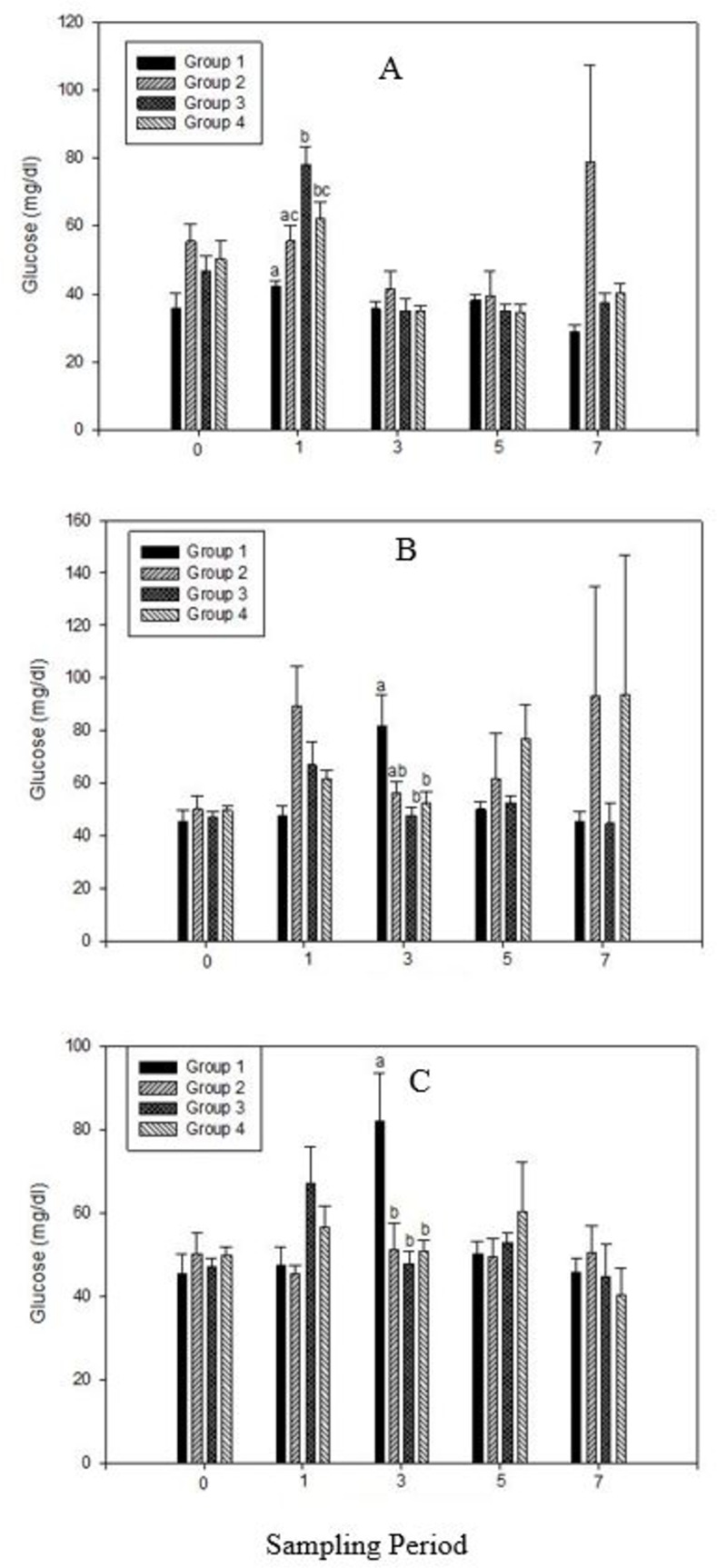
Analyses of blood glucose concentrations (mg/dl) in tilapia fed Vitamin C (A), Zinc (B), and combined Vitamin C and Zinc combined (C) supplemented feed. [In Fig 1A: Group 1: CFS, Group 2: VCS, Group 3: CFU, Group 4: VCU. Fig 1B: Group 1: CFS, Group 2: ZS, Group 3: CFU, Group 4: ZU. Fig 1C: Group 1: CFS, Group 2: VCZS, Group 3: CFU, Group 4: VCZU]. Data means ± SEM. Different alphabets indicate significant difference at P<0.05.

Fish sampling via MS-222 (Tricaine methanesulfonate) can induce stress due to the processes of chasing, capturing, and exposure to an unfamiliar environment [17, 27]. Despite these challenges, our study successfully induced stress in the lab, as evidenced by elevated blood glucose concentrations in the control feed groups compared to the supplemented groups during the initial stages of all three supplementation studies. In the analysis of blood glucose levels, no significant differences (P>0.05) were observed between the experimental groups receiving vitamin C (VC) supplementation at weeks 0, 3, 5, and 7 (Fig 1A). However, at week 1, significant differences (P<0.05) were noted between two of the sampled groups, where elevated blood glucose levels were observed in the CFS and CFU groups supplemented with Z (Fig 1B). Specifically, at week 3, the CFS group exhibited significantly higher blood glucose levels (P<0.05) compared to the CFU and ZU groups. At other sampling points, there were no significant differences in blood glucose levels with Z supplementation.

Regarding the combined VCZ supplementation (Fig 1C), at week 3, blood glucose concentrations were significantly elevated (P<0.05) in the CFS group, while the groups supplemented with VCZ had blood glucose concentrations similar to the CFU group. Elevated blood glucose concentrations are indicative of stress in fish [16, 28, 29].

**Fig 2:**
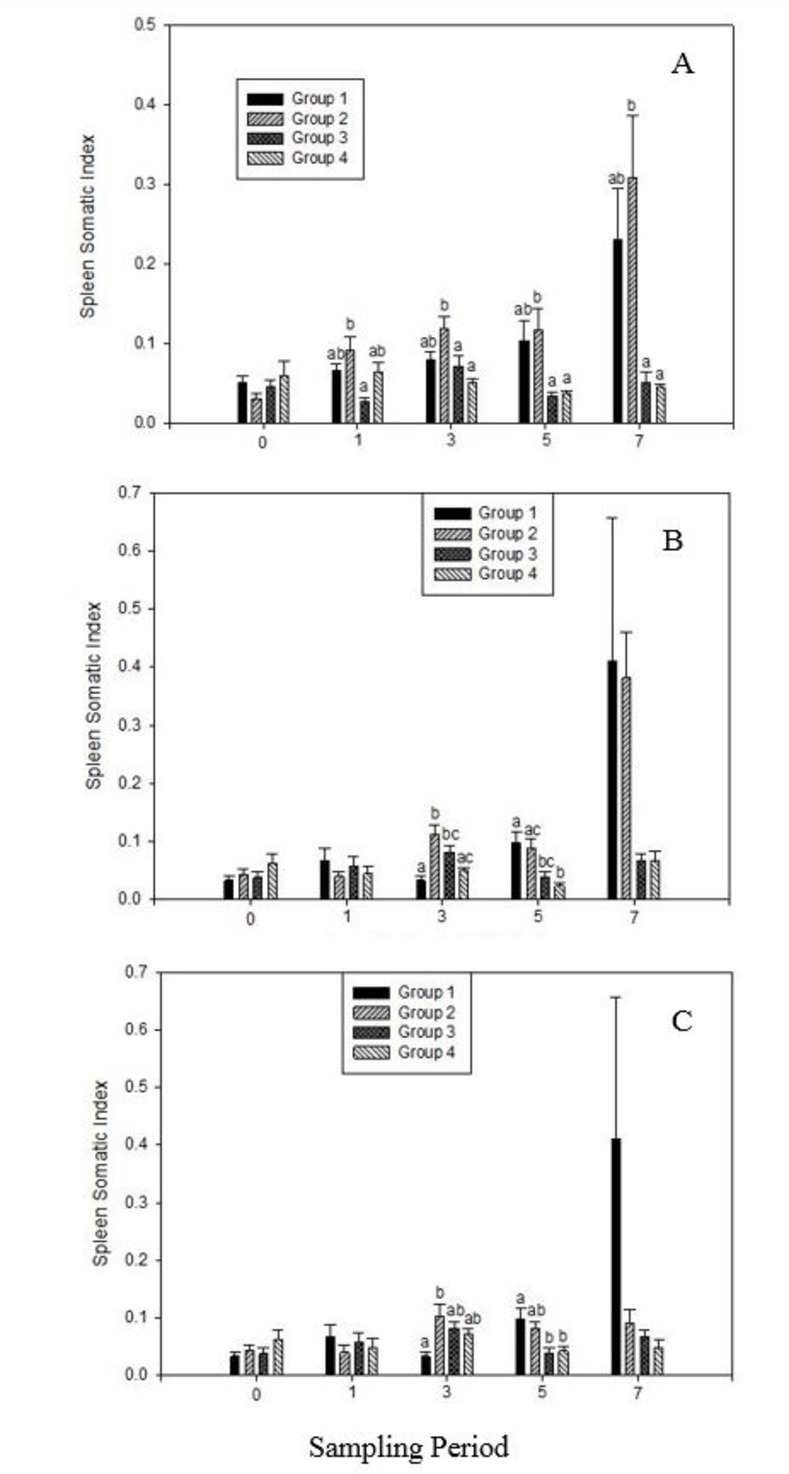
Analyses of spleen somatic indices (SSI) in tilapia fed Vitamin C (A), Zinc (B), and combined Vitamin C and Zinc combined (C) supplemented feed. [In Fig 2A: Group 1: CFS, Group 2: VCS, Group 3: CFU, Group 4: VCU. Fig 2B: Group 1: CFS, Group 2: ZS, Group 3: CFU, Group 4: ZU. Fig 2C: Group 1: CFS, Group 2: VCZS, Group 3: CFU, Group 4: VCZU]. Data means ± SEM. Different alphabets indicate significant difference at P<0.05.

In the context of SSI measurements with vitamin C (VC) supplementation (Fig 2A), there were significant differences (P<0.05) between CFU and CFS at weeks 1, 3, 5, and 7. Specifically, at weeks 3, 5, and 7, VCS exhibited higher SSIs (P<0.05) compared to CFU. Additionally, VCS showed significantly higher SSI (P<0.05) than VCU at weeks 3, 5, and 7. With zinc (Z) supplementation (Fig 2B), significant differences (P<0.05) were observed between the sampled groups at weeks 3, 5, and 7 for SSIs. At week 3, ZS had higher SSI (P<0.05) than CFS, whereas CFS had higher SSI (P<0.05) than ZU at week 5. For combined VC and Z supplementation (VCZ) (Fig 2C), significant differences were noted at weeks 3 and 5 (P<0.05). At week 3, VCZS showed significantly higher SSI (P<0.05) than CFS. At week 5, CFS exhibited significantly higher SSI (P<0.05) than the unstressed groups (VCZU and CFU).

**Fig 3:**
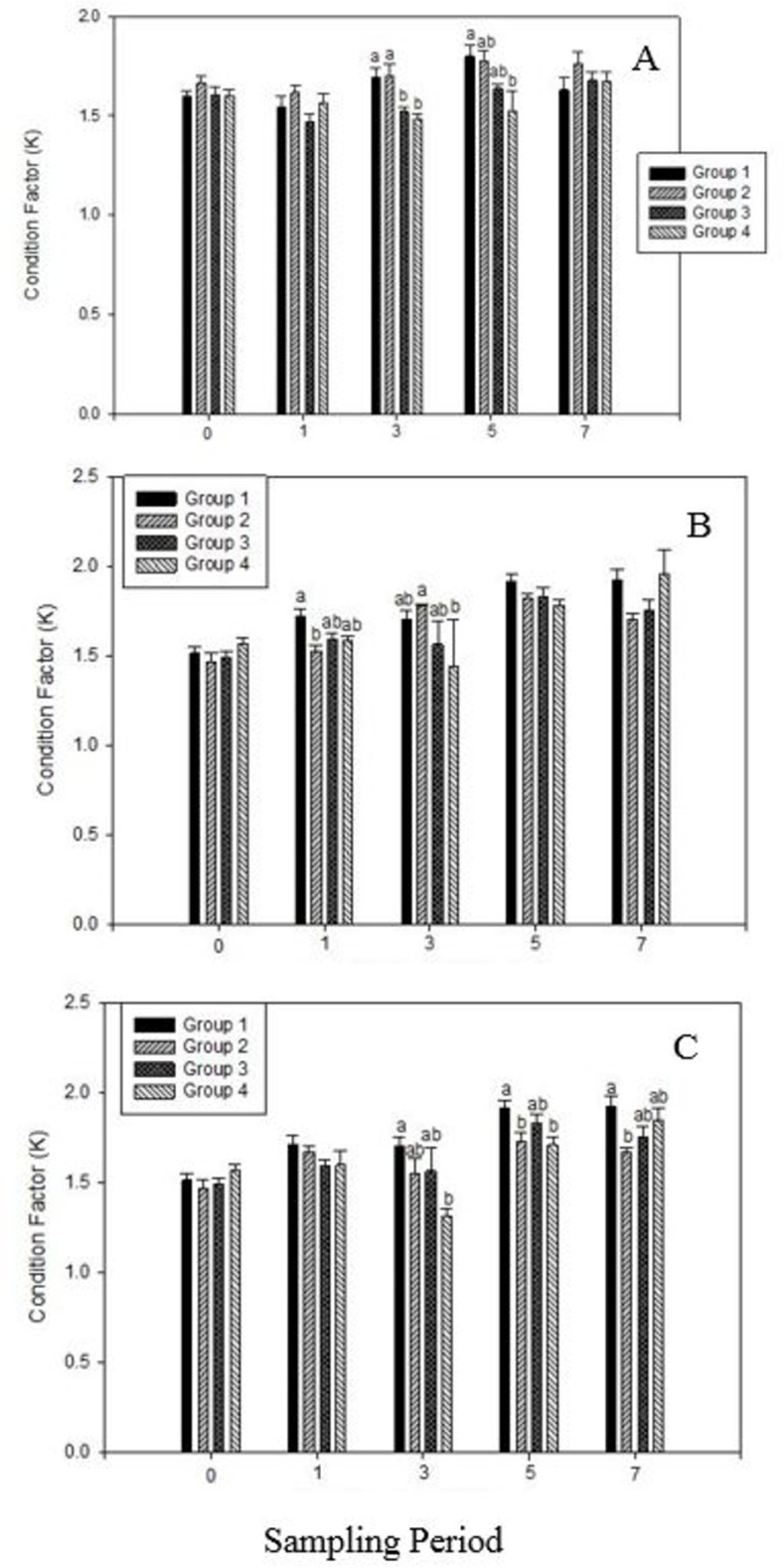
Analyses of condition factors in tilapia fed Vitamin C (A), Zinc (B), and combined Vitamin C and Zinc combined (C) supplemented feed. [In Fig 3A: Group 1: CFS, Group 2: VCS, Group 3: CFU, Group 4: VCU. Fig 3B: Group 1: CFS, Group 2: ZS, Group 3: CFU, Group 4: ZU. Fig 3C: Group 1: CFS, Group 2: VCZS, Group 3: CFU, Group 4: VCZU]. Data means ± SEM. Different alphabets indicate significant difference at P<0.05.

In the context of condition factor (K), no significant differences were observed within the sampled groups at weeks 0, 1, and 7 for VC supplementation (Fig 3A). However, significant differences (P < 0.05) in the mean K of the sampled fish were evident at weeks 3 and 5. Specifically, at week 3, both cool water groups (CFU and VCS) exhibited superior K values compared to both warm water groups (CFS and VCU). At week 5, CFU displayed a superior K value compared to VCU.

For Z supplementation (Fig 3B), no significant differences were noted at weeks 0, 5, and 7. Nonetheless, significant differences (P < 0.05) between the sampled groups were detected at weeks 1 and 3. At week 1, CFS had superior K values relative to VCS, and at week 3, VCS showed superior K values compared to VCU.

Regarding combined VCZ supplementation (Fig 3C), no significant differences were observed at weeks 0 and 1. However, significant differences (P < 0.05) were recorded at weeks 3, 5, and 7 among the sampled groups. At week 3, CFS exhibited a superior K value compared to VCZU. At week 5, CFS demonstrated superior K values compared to both VCZU and VCZS. At week 7, CFS had a superior K value relative to VCZS.

**Fig 4:**
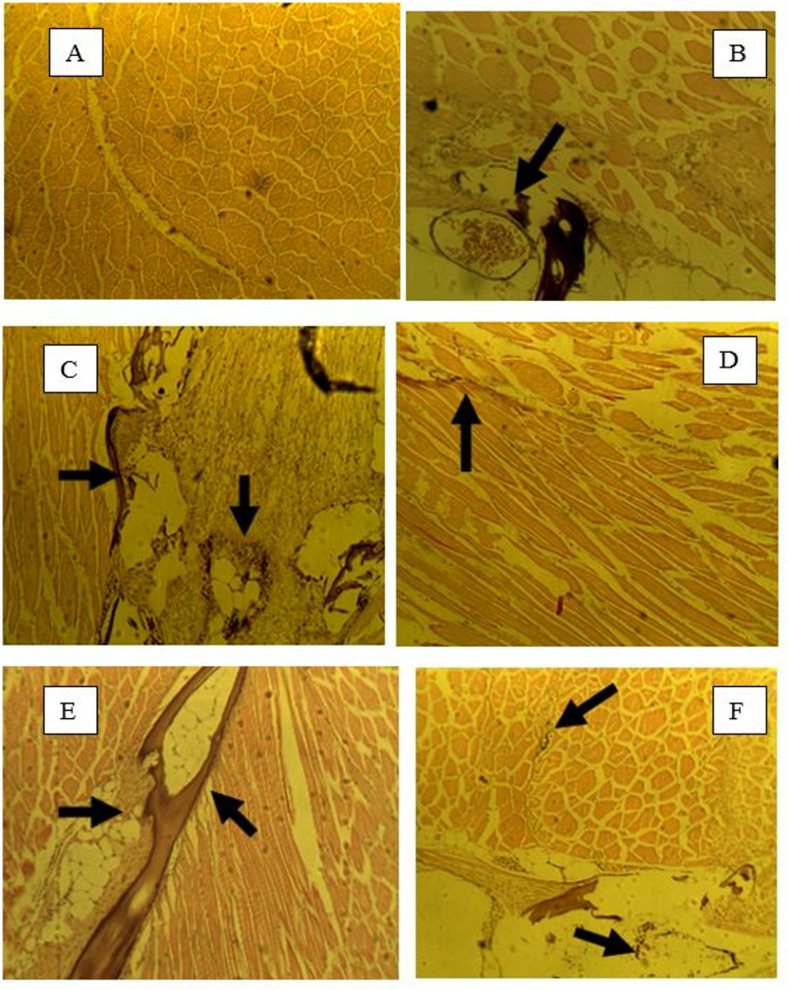
Histological sections of tilapia muscles in fish fed vitamin C supplemented feed. A: 4 hpi-CFS; B: 4 hpi-VCS; C: 8 hpi-CFS; D: 8 hpi-VCS; E: 24 hpi-CFS; F: 24 hpi-VCS. Arrows indicate the presence of cellular infiltrates.

Throughout the entire study period, the condition factors of all experimental groups were similar, indicating that cold water did not impede growth, contrary to previous research findings. Popma and Masser [2] reported that tilapia raised at optimal temperatures exhibited growth rates three times higher than those raised at 72°F. However, Mustafa et al. [32] demonstrated that tilapia experienced modest growth when reared at colder temperatures (60°F). Our findings, indicating that fish grown in colder temperatures and fed supplemented feed had similar condition factors to those grown at optimal temperatures, are consistent with the results of Mustafa et al. [32]. By the end of the study, no significant differences were observed between the stressed and unstressed groups. Images of slides from the control fish stressed (CFS) and supplemented feed stressed (VCS) groups are presented (Fig 4). After VC supplementation, no difference was observed between the tissues of fish from the CFU group and the control group at 4 hpi. However, while CFS tissues (Fig 4A) did not exhibit an immune response at 4 hpi, VCS tissues (Fig 4B) elicited an immune response characterized by the encapsulation of cellular infiltrates and thioglycolate droplets. CFU tissues showed a weakened immune response at 4 hpi, whereas VCU tissues elicited a noticeable immune response. At 8 hpi, all fish displayed immune responses. However, fish in the CFS group (Fig 4C) exhibited a stronger immune response than those in the CFU group, while both VCS (Fig 4D) and VCU groups displayed similar immune responses. At 24 hpi, CFU and CFS fish (Fig 4E) showed the presence of cellular components, whereas VCS fish (Fig 4F) did not display any signs of cellular infiltrates or tissue damage. By 24 hpi, both CFU and VCS groups were showing signs of tissue regeneration. In terms of the inflammatory response, VCS fish had a constant inflammatory response at 4, 8, and 24 hpi. In contrast, CFU fish had a consistent but relatively weaker response over the same periods. The VCU group exhibited a strong inflammatory response at 4 hpi, which gradually declined at 8 and 24 hpi.

**Fig 5:**
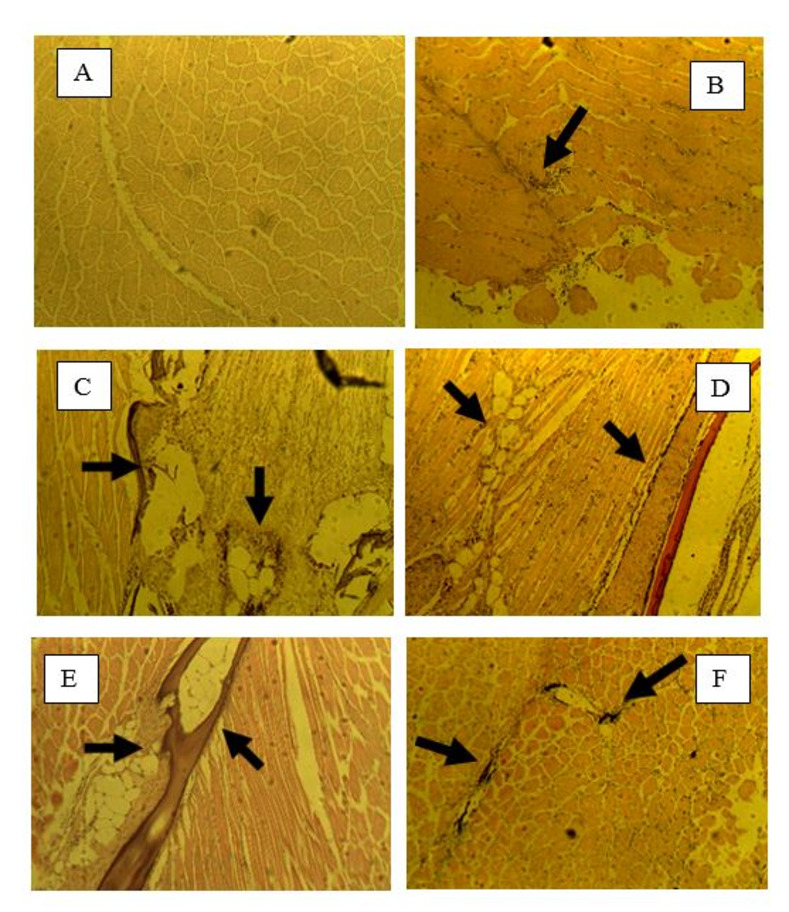
Histological sections of tilapia muscles in fish fed zinc supplemented feed. A: 4 hpi-CFS; B: 4 hpi-VCS; C: 8 hpi-CFS; D: 8 hpi-VCS; E: 24 hpi-CFS; F: 24 hpi-VCS. Arrows indicate the presence of cellular infiltrates.

With Z supplementation, a relative lack of inflammatory response was observed at 4 hours post-infection (hpi) (Fig 5A and 5B). By 8 hpi, a stronger inflammatory response was evident across all feed groups (Fig 5C and 5D). At 24 hpi, the CFU group exhibited a weaker inflammatory response, while the other feed groups continued to show a strong inflammatory response (Fig 5E and 5F). Fish from the CFS group elicited a latent inflammatory response, which became apparent by 8 hpi, with a high degree of cellular infiltrates accumulation maintained from 8 hpi through to 24 hpi. Both VS and VU groups displayed strong inflammatory responses throughout all sampling periods.

**Fig 6:**
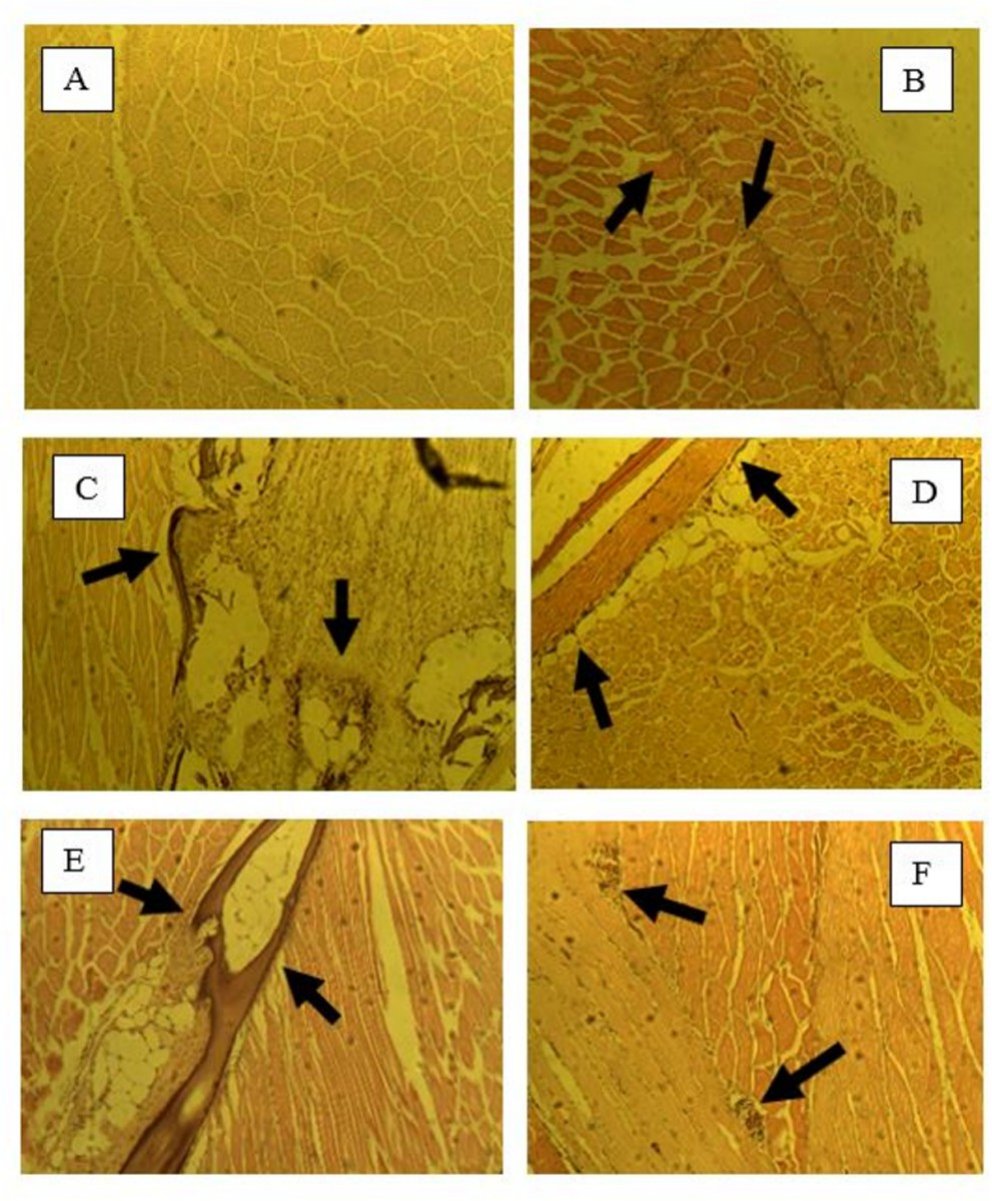
Histological sections of tilapia muscles in fish fed vitamin C and zinc combined supplemented feed. A: 4 hpi-CFS; B: 4 hpi-VCS; C: 8 hpi-CFS; D: 8 hpi-VCS; E: 24 hpi-CFS; F: 24 hpi-VCS. Arrows indicate the presence of cellular infiltrates.

With combined VCZ supplementation, a strong inflammatory response was observed in the supplemented feed groups at 4 hpi, compared to the control feed groups (Fig 6A and 6B). By 8 hpi, both control feed groups exhibited inflammatory responses, along with the supplementary feed groups (Fig 6C and 6D). Muscle tissues in the supplementary feed groups showed signs of regeneration. At 24 hpi, the VCZU group had ceased producing an immune response and the tissue appeared regenerated, whereas the VCZS and control groups still had cellular infiltrates (Fig 6E and 6F).

In most cases, fish fed with commercial feed exhibited a response by 8 hpi, as histological sections indicated that CFS fish elicited a latent inflammatory response by 8 hpi. Fish in the CFU groups exhibited an immediate inflammatory response and cellular infiltrate accumulation at 4 hpi. In contrast, VCZ co-supplemented fish elicited an immediate inflammatory response post-injection. At 8 hpi, no damaged muscle was evident in fish held in the warm water tank, as observed in slides obtained from VCZ co-supplemented fish. The effects of regenerated muscle cells were also evident at 24 hpi, indicating the powerful synergistic effects of vitamin C and zinc as immune boosters. Research by Kraus et al. [33] has shown that supplementation with antioxidants such as vitamin C significantly improved osmotic fragility in zinc-deficient rats without affecting zinc concentrations. Furthermore, it was determined that antioxidants decreased oxidative damage in erythrocytes of zinc-deficient rats. These results further illustrate the positive effects of vitamin C and zinc supplementation in feeds. VC supplementation aided in muscle regeneration after damage by 24 hpi, as observed with the VCU group. Although zinc supplementation alone did not lead to muscle cell regeneration by 24 hpi, the combination of vitamin C and zinc (VCZ) greatly improved wound healing, as no damage was evident at 8 and 24 hpi in VCZ fish.

## Discussion

Our study demonstrated that the control feed groups exhibited elevated blood glucose concentrations during the initial stages of supplementation, indicating that these groups were subjected to a significant stress response. The absence of significant differences in blood glucose levels between the supplemented and control groups at most sampling points suggests that vitamin C and zinc supplementation may play a role in mitigating the effects of stress, particularly during the early stages of chronic stress.

In addition to endocrine and cellular markers for stress, spleen size in fish is used as a predictor of disease resistance [28, 29]. The spleen filters blood, removing damaged or dying cells and foreign organisms [8, 30, 31]. A smaller spleen indicates stress and reduced disease resistance due to decreased blood circulation through the spleen [28, 29, 31]. Our experiments demonstrated that the stressed groups had lower SSIs, indicative of stress, with gradual decreases by week 7 across all studies. This indicates the successful simulation of stress conditions and acclimatization of the fish to the experimental conditions.

The condition factor (K) analysis also revealed interesting insights. The lack of significant differences in K values across the groups, except at certain weeks, suggests that supplementation with vitamin C and zinc did not adversely affect the growth of fish. The superior K values observed in the cool water groups at specific time points could be attributed to the positive effects of these supplements in cooler temperatures, as noted in previous studies [2, 32].

The histological analysis provided further evidence of the beneficial effects of supplementation. The immune response observed in the supplemented groups, particularly those receiving both vitamin C and zinc, was more robust compared to the control groups. This was evident in the reduced presence of cellular infiltrates and tissue damage at later time points, indicating that the combined supplementation enhanced the fish’s ability to recover from stress and infection.

The findings of this study are consistent with previous research on the stress-mitigating effects of vitamin C and zinc supplementation. Kraus et al. [33] showed that vitamin C supplementation significantly improved the stress resilience of zinc-deficient animals, which aligns with our observation of stable blood glucose levels and improved SSI in the supplemented groups. However, our results contrast with the study by Popma and Masser [2], who reported that tilapia growth was significantly reduced at suboptimal temperatures. In contrast, our study found that the condition factors of fish reared at cooler temperatures with supplementation were comparable to those raised at optimal temperatures, indicating that these supplements may help offset the adverse effects of colder environments. Mustafa et al. [32] also noted modest growth in tilapia at lower temperatures, supporting our conclusion that vitamin C and zinc can play a crucial role in maintaining fish health and growth under such conditions.

The results of this study have practical implications for tilapia farming, especially in regions where water temperatures are lower than the species’ optimal range. The ability of vitamin C and zinc to stabilize blood glucose levels, reduce spleen size reduction, and maintain condition factors suggests that these supplements could be vital in improving the resilience of tilapia to stressors associated with cold water. Farmers in cooler climates could benefit from incorporating these supplements into their feeding regimes, as they may help reduce mortality rates, improve growth performance, and enhance the overall health of the fish. Based on our findings, we recommend that tilapia farmers in colder regions consider using vitamin C and zinc supplementation as a strategy to mitigate the negative impacts of cold stress and improve the productivity of their aquaculture operations.

## Conclusions

Overall, our findings suggest that supplementation with vitamin C and zinc can mitigate the physiological stress response in fish, as indicated by the parameters checked in this study. The histological analysis further confirmed that these supplements enhance the immune response, leading to better recovery and tissue regeneration after stress-induced damage. This study contributes to the growing body of evidence supporting the use of dietary supplements to improve overall health in aquaculture species. Additionally, the study provides practical recommendations for tilapia farming under cold water conditions, offering a potential strategy to improve the sustainability and profitability of tilapia aquaculture in cooler climates.

Future studies will investigate the effects of VC and Z supplementation by completely removing these nutrients from the control fish feed. However, this study illustrates that increasing VC and Z supplementation in commercial fish feed enhances wound healing in Nile Tilapia (*O*. *niloticus*).

## Supporting information

S1 Dataset (XLSX)
